# *In vitro *anthelmintic activity of *Pothomorphe umbellata *(L.) Miq. (Piperaceae) against gastrointestinal parasites from sheep

**DOI:** 10.1186/1753-6561-8-S4-P155

**Published:** 2014-10-01

**Authors:** Luis Eduardo Ferreira, Pedro Castro, Franca Suzelei, Beleboni Rene

**Affiliations:** 1 Unit of Biotechnology, Ribeirão Preto University (UNAERP), Ribeirão Preto, SP, Brazil

## 

Sheep farming in Brazil has been proved to be a promising economical activity by the offering of products with increased interest. Infections with gastrointestinal parasites from family of Trichostrongylidae, especially represented by *Haemonchus contortus*, are one the most limiting problem in sheep production, aggravated by the increasing resistance of nematodes to traditional anthelmintic drugs. Ethnopharmacological data have indicated the *Pothomorphe umbellata *as a promising alternative for the control of gastrointestinal nematodes. The aim of this work was to evaluate the *in vitro *anthelmintic effects of *P. umbellata *aqueous leaf extract against eggs, infective larvae and adult forms of parasitic nematodes from Trichostrongylidae family from sheep. At higher doses, *P. umbellata *extract showed 95.82%, and 32.18% of efficacy in egg hatch test (EHT) and larval motility test (LMT), respectively. In the adult worm motility test, worms were completely immobilized within the first 4-6 hours of nematode exposition to different dilutions of extract. Thus, these data showed that the evaluated extract presented significant anthelmintic activity, which are of high biotechnological interest and useful towards the growth of sheep farming in Brazil.
